# Knockdown of amyloid precursor protein increases calcium levels in the endoplasmic reticulum

**DOI:** 10.1038/s41598-017-15166-2

**Published:** 2017-11-06

**Authors:** Kinga Gazda, Jacek Kuznicki, Tomasz Wegierski

**Affiliations:** Laboratory of Neurodegeneration, International Institute of Molecular and Cell Biology, 02-109 Warsaw, Poland

## Abstract

Familial Alzheimer’s disease (AD) is caused by mutations in the genes that encode amyloid precursor protein (APP) and presenilins. Disturbances in calcium homeostasis have been observed in various cellular and animal models of AD and are proposed to underlie the pathogenesis of the disease. Furthermore, wildtype presenilins were shown to regulate endoplasmic reticulum (ER) calcium homeostasis, although their precise mechanism of action remains controversial. To investigate whether APP also affects ER calcium levels, we used RNA interference to target the APP gene in cultured T84 cells in combination with two types of ER calcium sensors. Using a genetically encoded calcium indicator, GEM-CEPIA1er, we found that APP-deficient cells exhibited elevated resting calcium levels in the ER and prolonged emptying of ER calcium stores upon the cyclopiazonic acid-induced inhibition of sarco-endoplasmic reticulum calcium-ATPase. These effects could be ascribed to lower ER calcium leakage rates. Consistent with these results, translocation of the endogenous ER calcium sensor STIM1 to its target channel Orai1 was delayed following ER calcium store depletion. Our data suggest a physiological function of APP in the regulation of ER calcium levels.

## Introduction

Calcium (Ca^2+^) is a versatile cellular second messenger^1^. It plays an important role in a multitude of cellular activities, ranging from gene transcription to neurotransmission. Inside the cell, Ca^2+^ ions are predominantly sequestered in the endoplasmic reticulum (ER). The steep gradient of Ca^2+^ concentrations between the cytosol and ER is maintained by sarco-endoplasmic reticulum Ca^2+^ adenosine triphosphatase (SERCA) pump^1^. In resting cells, the activity of SERCA is counteracted by poorly defined Ca^2+^-conducting passive leak channels^2^. Upon cell stimulation, Ca^2+^ that is stored in the ER is released into the cytosol through the activity of inositol triphosphate-3 (IP_3_) receptors and ryanodine receptors^1^. The resulting drop in ER Ca^2+^ concentrations ([Ca^2+^]_ER_) is sensed by stromal interaction molecule 1 (STIM1), an integral ER membrane protein^3^. The dissociation of Ca^2+^ from its EF-hand motif results in STIM1 oligomerisation and translocation toward ER-plasma membrane junctions where it binds and activates Orai Ca^2+^ channels^3^. The subsequent Ca^2+^ influx is referred to as store-operated calcium entry (SOCE), which both refills Ca^2+^ stores and sustains Ca^2+^ signalling^4–6^. Orai channels are composed of homologous Orai1-3 proteins, from which Orai1 contributes most to SOCE in different cell types^7^. Moreover, the interaction between the ER Ca^2+^ sensor STIM1 and Orai1-based Ca^2+^ channels has been demonstrated to be sufficient for SOCE^8^.

The dysregulation of Ca^2+^ homeostasis has been proposed to underlie various pathological conditions, such as neurodegenerative disorders, including incurable Alzheimer’s disease (AD)^9,10^. Most AD cases are sporadic and affect elderly people, but some cases (1–6%) have an early-onset and are caused by mutations in the genes that encode presenilin-1 (PS1), presenilin-2 (PS2), and amyloid precursor protein (APP)^11^. Although such familial AD (FAD) cases are relatively rare, the disease-linked proteins have been intensively studied to elucidate the pathogenesis of AD. Most FAD-causing mutations map to PS1, the enzymatic component of the γ-secretase proteolytic complex^12^. PS1 FAD mutations have been repeatedly shown to enhance ER Ca^2+^ signalling in patient cells and various cellular and animal disease models, supporting the “calcium hypothesis” of AD^13,14^. The expression of FAD-causing PS1 mutants also reduces SOCE, whereas the downregulation of PS1 or inhibition of γ-secretase activity enhances SOCE^14^. However, still debatable is whether PS1 affects SOCE machinery directly or only indirectly by altering ER Ca^2+^ content^15^. The precise effects of presenilins (PSs) and PS FAD mutations on ER Ca^2+^ levels are also disputed because measurements of [Ca^2+^]_ER_ with the help of ER-targeted indicators have yielded contradictory results^16–24^. Consequently, several different mechanisms have been proposed to explain the role of PS FAD mutations in the observed enhancement of ER Ca^2+^ signalling^16,17,22,25^.

Even less is known about the role of APP in ER Ca^2+^ homeostasis. APP is a single-pass transmembrane protein that undergoes sequential proteolytic cleavage^26^. Amyloidogenic processing is performed by β- and γ-secretases, which liberate two short fragments from the APP molecule: β-amyloid and APP intracellular C-terminal domain (AICD). β-amyloid peptides may lead to an elevation of cytosolic Ca^2+^ levels by activating Ca^2+^ influx mechanisms or forming Ca^2+^-permeable pores themselves^10^. AICD was shown to be required for bradykinin-evoked ER Ca^2+^ release in fibroblasts^27^. However, the Ca^2+^-related functions of APP-derived fragments were inferred solely from changes in cytosolic Ca^2+^ levels. In contrast, using both cytosolic and ER-targeted Ca^2+^ indicators, Oules *et al*. recently reported that the overexpression of a FAD-causing APP_SWE_ mutant reduced ER Ca^2+^ load capacity through higher activity of Ca^2+^ release mechanisms from this organelle^28^. In the present study, we used a different approach to shed light on the physiological function of APP and examined ER Ca^2+^ levels in cells with downregulated *APP* expression. For this purpose, we used both the ER-targeted genetically encoded Ca^2+^ indicator (GECI) GEM-CEPIA1er^29^ and the endogenous ER Ca^2+^ sensor STIM1. We found that APP-deficient cells had elevated resting levels of Ca^2+^ in the ER and exhibited delayed translocation of STIM1 to Orai1 upon ER Ca^2+^ store depletion. Our data suggest a regulatory role for APP in ER Ca^2+^ homeostasis.

## Results

### Endogenous STIM1 co-localises with Orai1 following CPA-induced ER Ca^2+^ store depletion in T84 cells

**Figure 1:**
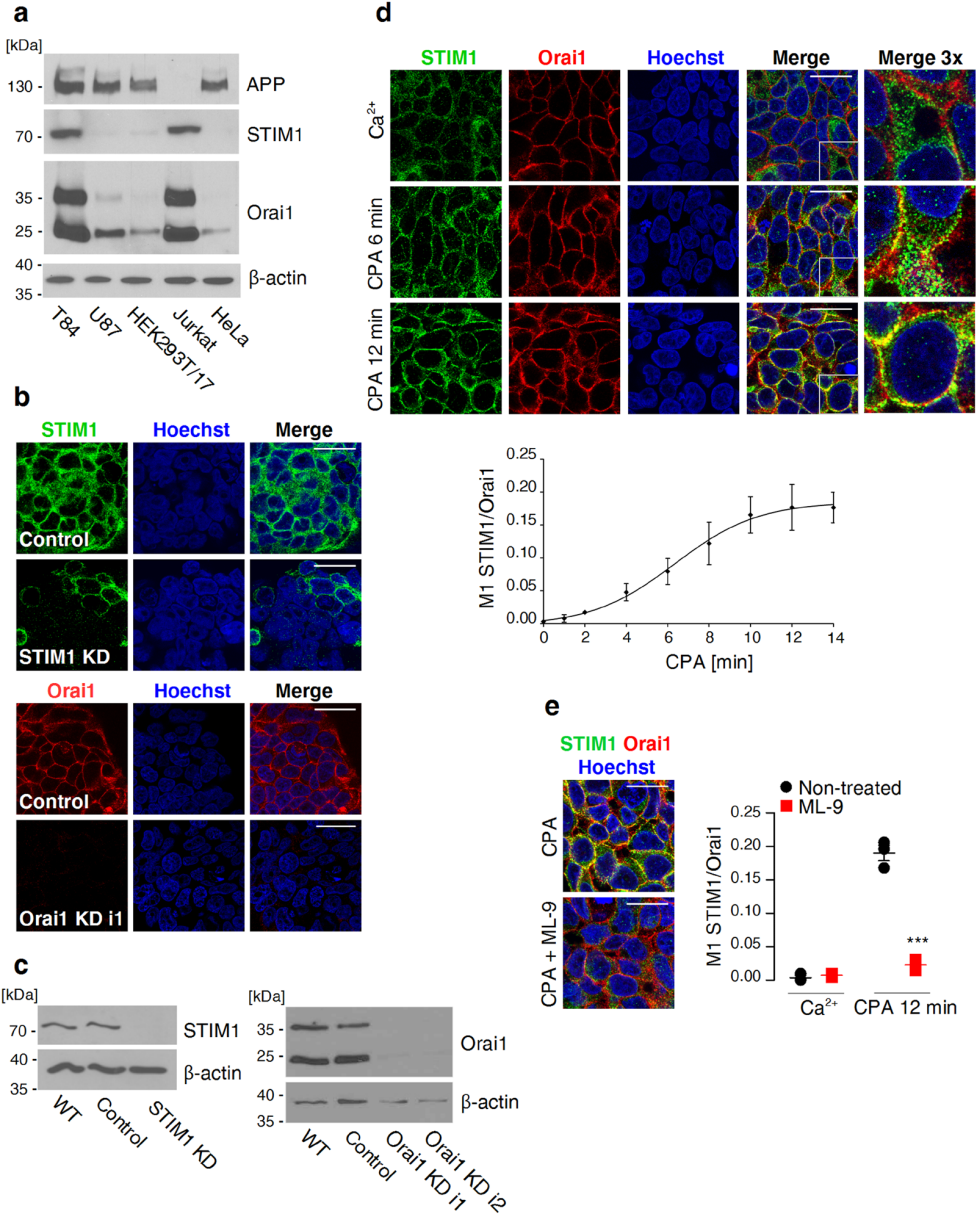
Analysis of the translocation of STIM1 to Orai1 by quantitative co-localisation. (**a**) Immunoblots of endogenous APP, STIM1, and Orai1 in the indicated cell lines. β-actin was probed as a loading control. (**b**) T84 cells with *STIM1* or *ORAI1* gene expression knockdown (STIM1 KD and Orai1 KD i1, respectively) and T84 cells expressing control shRNA (Control) were stained with STIM1 Ab (green) or Orai1 Ab (red). Nuclei were stained with Hoechst 33342 (blue). Scale bar on merged images = 20 µm. (**c**) Western blot analysis of endogenous STIM1 (left) or Orai1 (right) in three lines of T84 cells with gene expression knockdown (STIM1 KD, Orai1 KD i1, and Orai1 KD i2) compared with cells expressing control shRNA (Control) and wildtype T84 cells (WT). β-actin was probed as a loading control. (**d**) Co-localisation of STIM1 with Orai1 upon ER Ca^2+^ store depletion with 30 µM CPA. T84 cells were fixed before (Ca^2+^ or time 0), or at indicated times after the addition of CPA in Ca^2+^-free buffer and stained with STIM1 Ab (green), Orai1 Ab (red), and Hoechst 33342 (blue). Yellow in the merged images indicates co-localisation between STIM1 and Orai1 (marked regions were magnified 3× and shown on the right). Scale bar = 20 µm. The curve shows coefficients of co-localisation of STIM1 with Orai1 (M1 STIM1/Orai1) as a function of time after the addition of CPA. The data were fitted to non-linear regression. The results are expressed as the mean ± SEM from four experiments. (**e**) ML-9 blocked the co-localisation of STIM1 with Orai1 in cells with depleted ER Ca^2+^ stores. Wildtype T84 cells, non-treated or pretreated with 50 µM ML-9 as indicated, were fixed before (Ca^2+^) or 12 min after the addition of CPA in Ca^2+^-free buffer. Images show merged signals of CPA-treated cells that were stained as in (d). Scale bar = 20 µm. Aligned dot plots show calculated mean coefficients of co-localisation of STIM1 with Orai1 (M1 STIM1/Orai1) ± SEM (*n* = 3). Differences between ML-9-treated and non-treated cells were analysed using unpaired *t*-tests (***p < 0.001).

During ER Ca^2+^ store depletion, STIM1 proteins oligomerise and translocate within ER membranes toward cell surface-localised Orai1 Ca^2+^ channels^3^. This process can be experimentally elicited by blocking SERCA with cyclopiazonic acid (CPA), which depletes Ca^2+^ from the ER through passive leakage. To investigate the potential effect of APP on the translocation of STIM1 to Orai1, we searched for a cell line that produces all three proteins at high levels. We confirmed substantial amounts of Orai1 in lysates from T84 and Jurkat cells as previously reported by others^30^. However, among the tested cell lines, only T84 cells satisfied our search criteria (Fig. 1a). Therefore, this cell line was used for the subsequent experiments. In T84 cells at rest, Orai1 localised to the cell boundaries, and the STIM1 signal concentrated around nuclei (Fig. 1b,d). The specificity of the antibodies was confirmed in T84 cells, in which *ORAI1* and *STIM1* expression was knocked down by RNA interference (RNAi) using virally-delivered specific short-hairpin RNA (shRNA) sequences (Fig. 1b,c). The kinetics of STIM1 translocation was then analysed by measuring the co-localisation of STIM1 and Orai1. Wildtype T84 cells were treated with CPA in Ca^2+^-free buffer, fixed at defined time-points after the addition of CPA, and immunolabelled. The fraction of STIM1 that specifically co-localised with Orai1 was calculated for each time-point (see Methods). Using this approach, a time-dependent increase in the fraction of STIM1 that was present with Orai1 was found, with half maximal co-localisation values reached 6.3 min after the addition of CPA (Fig. 1d). Pretreatment with ML-9, a SOCE inhibitor that acts by blocking STIM1 translocation^31^, dramatically reduced the co-localisation of STIM1 with Orai1 at 12 min, at which time STIM1 translocation reached saturation in ML-9-untreated cells (Fig. 1d,e). In contrast, two other widely used SOCE inhibitors, SKF-96365 ^32^ (a rather non-selective ion channel blocker) and YM-58483 ^33^ (or BTP2; a potent inhibitor of store-operated channels with a not fully understood mechanism of action^34^) did not prevent the translocation of STIM1 to Orai1 (measured at 12 min; Supplementary Fig. S1), as expected from blockers of Ca^2+^ entry. Interestingly, however, YM-58483 delayed the translocation, as indicated by the significantly reduced co-localisation of STIM1 with Orai1 at 6 min after the addition of CPA. Thus, YM-58483 appears to exert some effect on the STIM1-Orai1 coupling upon CPA-induced ER Ca^2+^ store depletion, at least at the relatively high dose used in our assay (10 µM). Altogether, these results indicate that T84 cells can be used to analyse the kinetics of endogenous STIM1 translocation toward native Orai1 channels by quantitative co-localisation.

### Resting levels of ER Ca^2+^ determine the efficiency of CPA-evoked STIM1 translocation to Orai1

**Figure 2:**
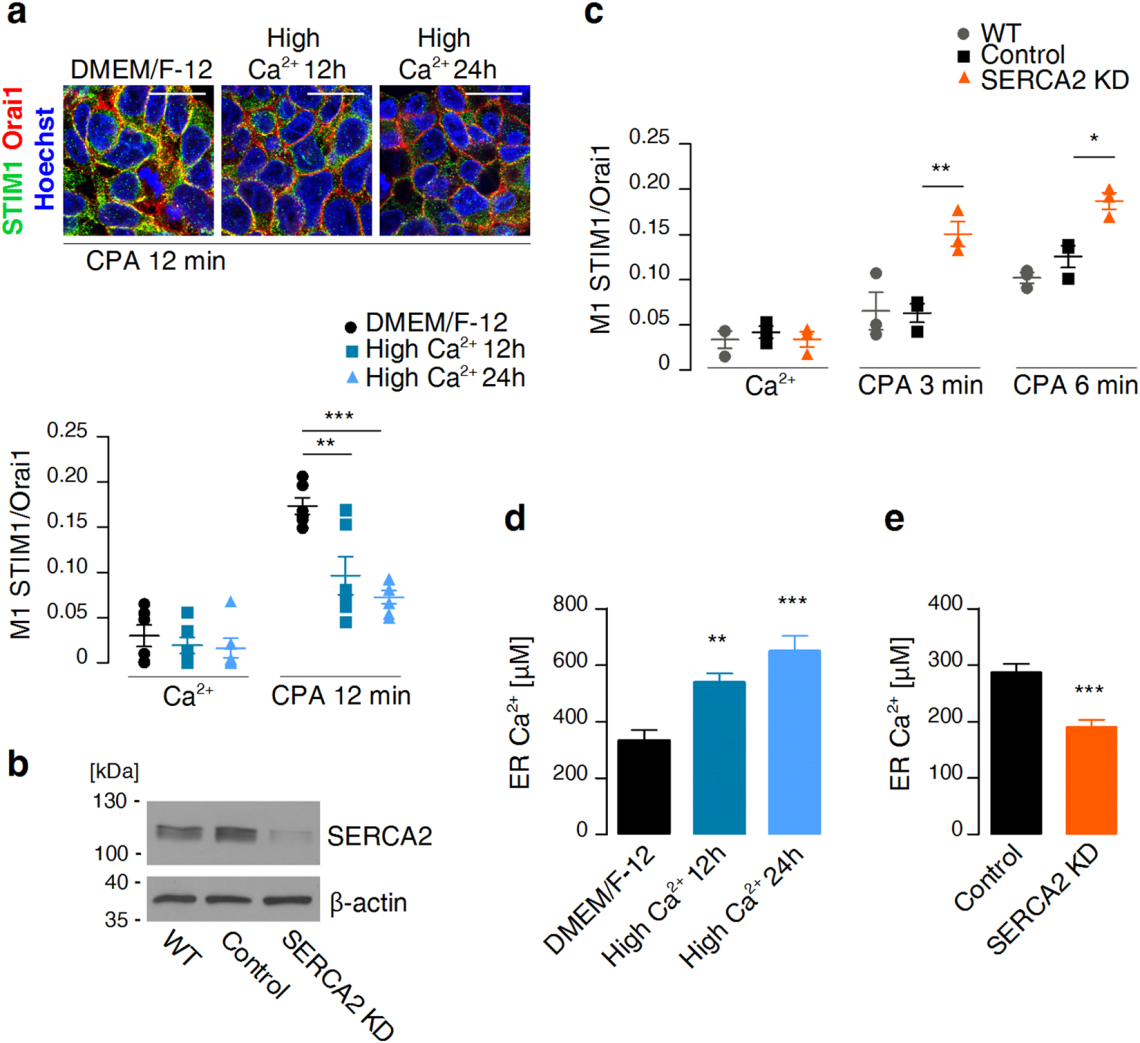
Modulation of ER Ca^2+^ content affects STIM1 translocation upon CPA-evoked store depletion. (**a**) Wildtype T84 cells were grown in normal media (DMEM/F-12) or in high-Ca^2+^ media for 12 or 24 h to raise intracellular Ca^2+^ content and then fixed before (Ca^2+^) or 12 min after the addition of CPA in Ca^2+^-free buffer. Images show merged signals of CPA-treated cells that were stained with STIM1 Ab (green), Orai1 Ab (red), and Hoechst 33342 (blue). Scale bar = 20 µm. Aligned dot plots show calculated mean coefficients of co-localisation of STIM1 with Orai1 (M1 STIM1/Orai1) ± SEM (*n* = 6). Differences from cells grown in normal media were analysed using unpaired *t*-tests (**p < 0.01, ***p < 0.001). (**b**) Western blot analysis of SERCA2 levels in T84 cells with gene expression knockdown (SERCA2 KD), T84 cells expressing control shRNA (Control), and wildtype T84 cells (WT). β-actin was probed as a loading control. (**c**) T84 cells expressing *SERCA2*-targeting shRNA (SERCA2 KD), T84 cells expressing control shRNA (Control), and wildtype T84 cells (WT) were fixed before (Ca^2+^), 3 min after, or 6 min after the addition of CPA in Ca^2+^-free buffer. Aligned dot plots show calculated mean coefficients of co-localisation of STIM1 with Orai1 (M1 STIM1/Orai1) ± SEM (*n* = 3). Differences from control cells were analysed using unpaired *t*-tests (*p < 0.05, **p < 0.01). (**d**) GEM-CEPIA1er-expressing T84 cells were grown in normal medium (DMEM/F-12; *n* = 6) or in high-Ca^2+^ medium for 12 h (*n* = 6) or 24 h (*n* = 6) and imaged under a fluorescence microscope. Mean ER Ca^2+^ concentrations are shown as bars with standard errors. Differences from cells grown in normal media were analysed using unpaired *t*-tests (**p < 0.01, ***p < 0.001). (**e**) GEM-CEPIA1er signals of T84 cells expressing *SERCA2*-targeting shRNA (SERCA2 KD; *n* = 14) or control shRNA (Control; *n* = 15) were acquired under a fluorescence microscope. Mean ER Ca^2+^ concentrations are shown as bars with standard errors. Differences from control cells were analysed using unpaired *t*-tests (***p < 0.001).

As the next step, the ER Ca^2+^ content in T84 cells was experimentally modulated to evaluate the effect of initial [Ca^2+^]_ER_ on subsequent CPA-evoked STIM1 translocation to Orai1. T84 cells were first grown for 12 or 24 h in media that contained 10 mM Ca^2+^ (high Ca^2+^ medium) to increase intracellular [Ca^2+^], including [Ca^2+^]_ER_. The co-localisation of STIM1 with Orai1, measured 12 min after the addition of CPA in Ca^2+^-free buffer, was significantly reduced in these cells compared with control cells that were grown in normal media (Fig. 2a). To achieve the opposite effect (i.e., the reduction of initial [Ca^2+^]_ER_), the expression of *SERCA2* was downregulated by RNAi using a specific shRNA sequence (Fig. 2b). Acute inhibition of the remaining SERCA with CPA in these cells resulted in significant enhancement of the co-localisation of STIM1 with Orai1 both 3 and 6 min after the addition of CPA compared with T84 cells that carried control shRNA (Fig. 2c). Subsequently, the intended effects of the applied treatments on initial [Ca^2+^]_ER_ were confirmed in T84 cells that were transduced with lentiviruses that carried GEM-CEPIA1er, a novel genetically encoded ER Ca^2+^ indicator^29^. We chose GEM-CEPIA1er over the more-established D1ER^35^ because it has a higher apparent dissociation constant for Ca^2+^ and a much larger dynamic range. Thus, GEM-CEPIA1er was expected to detect [Ca^2+^]_ER_ more reliably than D1ER^36^. Calibration of the GEM-CEPIA1er signals indicated a significant increase in [Ca^2+^]_ER_ in cells that were grown in high Ca^2+^ media (mean ± SEM: 537.4 ± 32.5 µM for 12-h treatment and 647.6 ± 55.4 µM for 24-h treatment *vs*. 331.5 ± 38.8 µM for cells that were grown in normal media; Fig. 2d) and significant decrease in [Ca^2+^]_ER_ in cells with *SERCA2*-targeting shRNA (190.9 ± 12.2 µM *vs*. 287.1 ± 14.6 µM for cells with control shRNA; Fig. 2e). These results indicate that an increase in resting [Ca^2+^]_ER_ results in less efficient STIM1 translocation to Orai1, and a decrease in [Ca^2+^]_ER_ results in more efficient STIM1 translocation to Orai1 upon ER Ca^2+^ store depletion with CPA.

### APP-deficient cells exhibit elevated resting levels of ER Ca^2+^ and delayed CPA-evoked STIM1 translocation to Orai1

**Figure 3:**
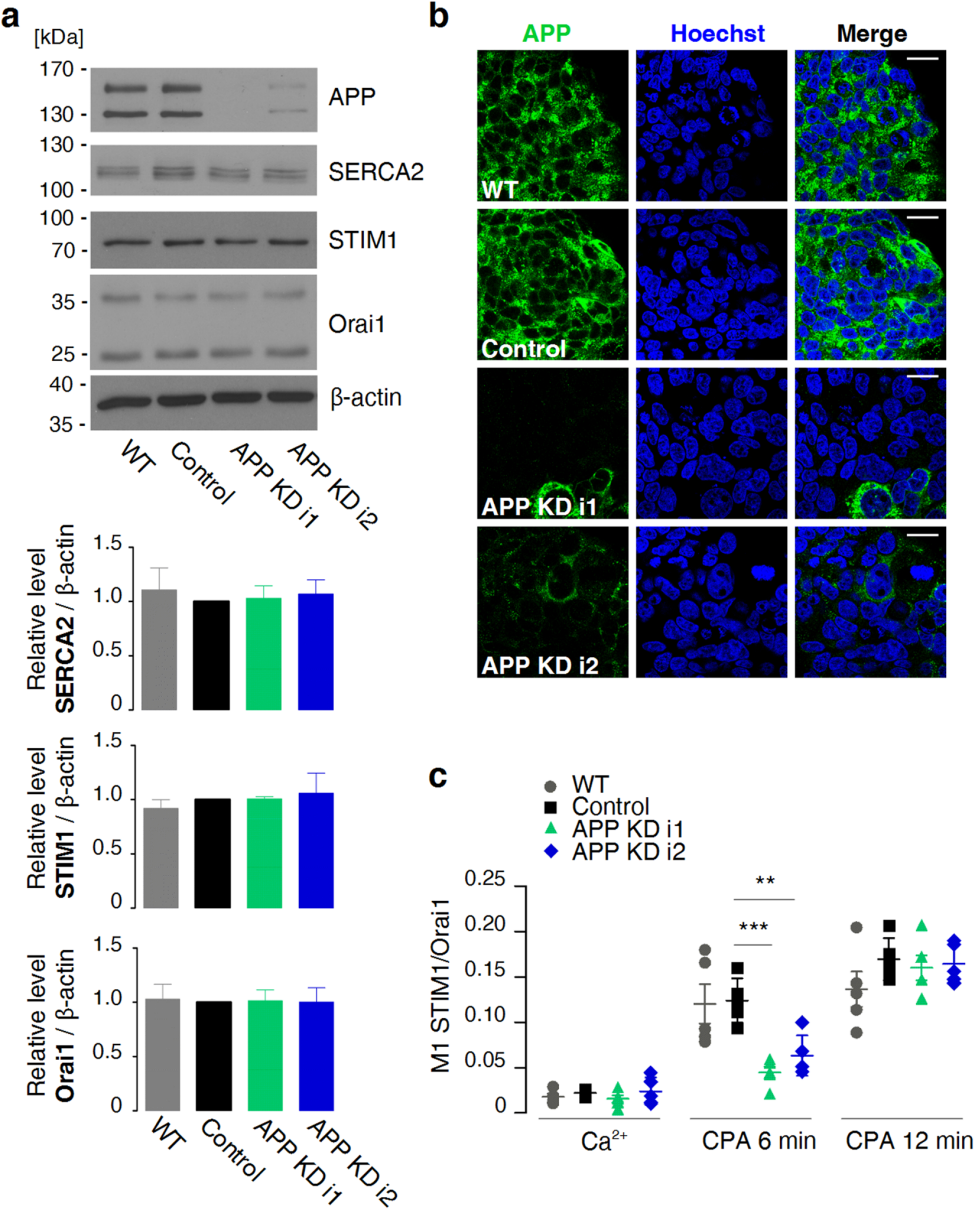
CPA-evoked translocation of STIM1 to Orai1 is delayed in APP-deficient T84 cells. (**a**) Western blot analysis of APP, SERCA2, STIM1, and Orai1 levels in T84 cells carrying *APP*-targeting shRNAs (APP KD i1 or APP KD i2), T84 cells carrying control shRNA (Control), and wildtype T84 cells (WT). The analysed protein signals were quantified relative to β-actin. The mean values were calculated from three cell lysates and are shown as bars with standard errors. A reference level of 1 was set for control cells, and differences from the reference level were analysed using one-sample *t*-tests. (**b**) Immunofluorescence staining of the aforementioned cells with APP Ab (green) and Hoechst 33342 (blue). Scale bar on merged images = 20 µm. (**c**) Cells as above were fixed before (Ca^2+^), 6 min after, or 12 min after the addition of CPA in Ca^2+^-free buffer. Aligned dot plots show calculated mean coefficients of co-localisation of STIM1 with Orai1 (M1 STIM1/Orai1) ± SEM (*n* = 5). Differences from control cells were analysed using unpaired *t*-tests (**p < 0.01, ***p < 0.001).

**Figure 4:**
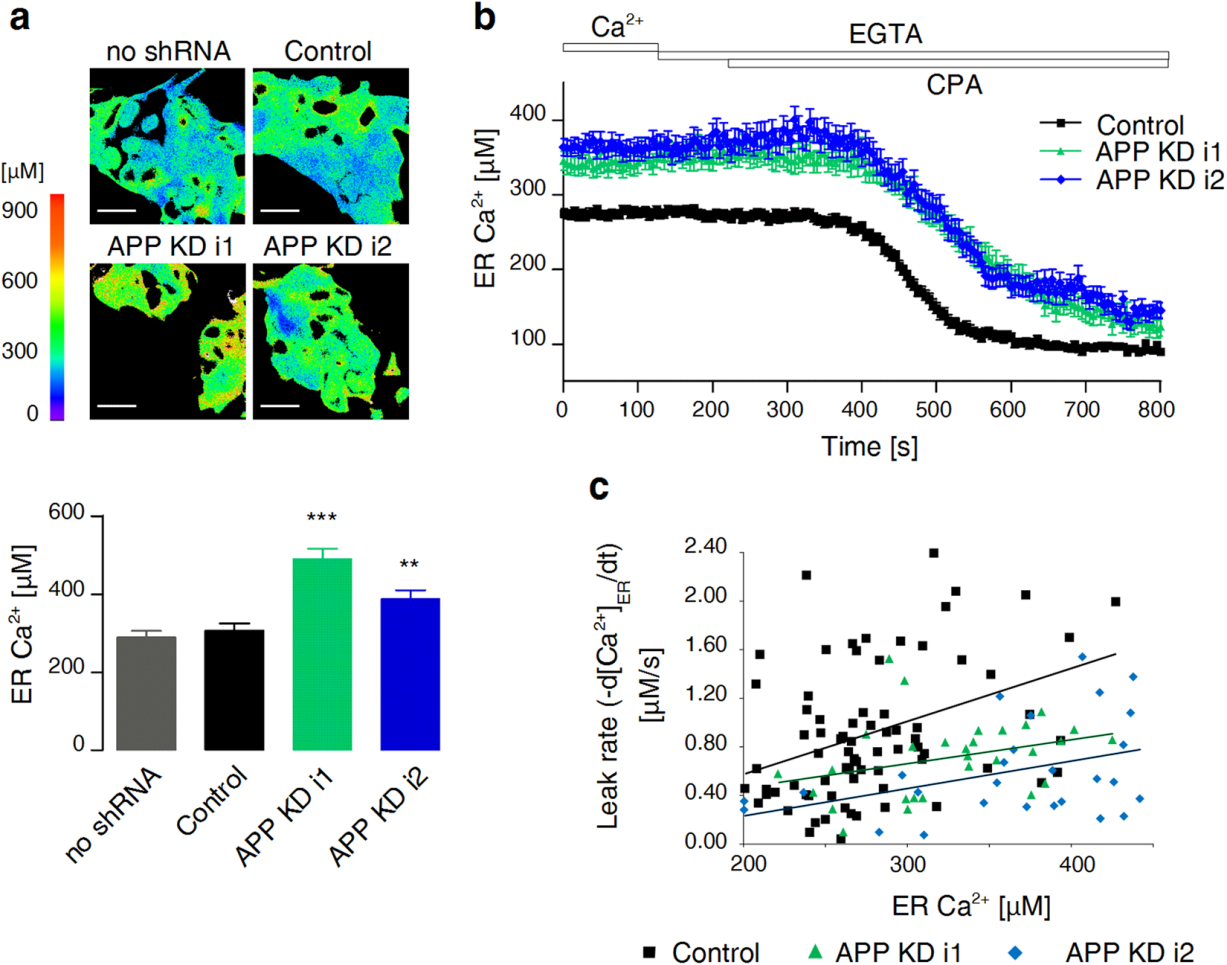
APP-deficient T84 cells have elevated resting levels of ER Ca^2+^ and lower ER Ca^2+^ leakage rates. (**a**) GEM-CEPIA1er signals of T84 cells expressing *APP*-targeting shRNA (APP KD i1 or APP KD i2; *n* = 20 and 22, respectively), control shRNA (Control; *n* = 24), or an empty shRNA cassette (no shRNA; *n* = 23) were acquired under a fluorescence microscope. Images show representative heat maps of ER Ca^2+^ concentrations in the analysed cells (scale on the left). Scale bar = 20 µm. Mean ER Ca^2+^ concentrations are shown as bars with standard errors. Differences from control cells were analysed using unpaired *t*-tests (**p < 0.01, ***p < 0.001). (**b**) Cells expressing GEM-CEPIA1er and *APP*-targeting shRNA (APP KD i1 or APP KD i2) or control shRNA were perfused with Ca^2+^-free solution that contained 30 µM CPA to induce passive Ca^2+^ leakage from the ER. Mean traces of individual ER regions of interest (ROIs) are shown, which were collected from 5–6 independent measurements for each cell line. Standard errors are shown as error bars. (**c**) The plots show Ca^2+^ leakage rates as a function of the initial [Ca^2+^]_ER_ for the same ROIs as shown in (b). The calculated regression lines are indicated.

After establishing the procedure to measure STIM1 translocation to Orai1, we knocked down *APP* expression in T84 cells by RNAi. Two polyclonal APP-deficient cell lines were prepared by transduction with lentiviruses. Each cell line stably expressed either of two different *APP*-targeting shRNAs (Fig. 3a,b). The downregulation of *APP* expression did not affect the levels of SERCA2, STIM1, or Orai1 proteins (Fig. 3a). APP-deficient cells were subsequently analysed for potential changes in ER Ca^2+^ homeostasis. Compared with control shRNA-expressing T84 cells, APP-deficient cells presented a significant decrease in the extent of STIM1 co-localisation with Orai1 that was measured 6 min after the addition of CPA (Fig. 3c). In contrast, no differences in STIM1-Orai1 co-localisation coefficients were found at 12 min (Fig. 3c), at which time STIM1 translocation reached saturation in wildtype cells (Fig. 1d). To investigate whether the delayed STIM1 translocation that was observed at 6 min after the addition of CPA was caused by the initial elevation of [Ca^2+^]_ER_, direct measurements of resting [Ca^2+^]_ER_ were performed. To this end, new polyclonal cell lines were prepared by transduction with viruses that carried GEM-CEPIA1er and either of two *APP*-targeting shRNAs, control shRNA, or an empty shRNA cassette. This line of experiments indeed confirmed substantially and significantly elevated resting [Ca^2+^]_ER_ in APP-deficient cells (489.6 ± 26.2 µM for APP KD i1, 386.8 ± 22.9 µM for APP KD i2, 306.2 ± 18.3 µM for cells with control shRNA, 289.7 ± 16.1 µM for cells without shRNA; Fig. 4a). Similar results were obtained for HeLa cells that were transduced with the same set of viruses (621.5 ± 30.6 µM for APP KD i1, 549.8 ± 35.1 µM for APP KD i2, 417.7 ± 21.2 µM for cells with control shRNA, 363.7 ± 20.5 µM for cells without shRNA; Supplementary Fig. S2), indicating that the APP-mediated effect on ER Ca^2+^ is not exclusive to T84 cells. APP-deficient T84 cells also exhibited prolonged emptying of ER Ca^2+^ stores (i.e. the same descending [Ca^2+^]_ER_ values were reached at a later time than in control shRNA-expressing cells) when perfused with the CPA-containing Ca^2+^-free buffer (Fig. 4b). To determine whether APP-deficient cells had altered activity of leak channels, the rates of decreases in [Ca^2+^]_ER_ in individual CPA-perfused cells were calculated. The leakage rates apparently depended on the initial levels of ER Ca^2+^ in each cell (Fig. 4c). Therefore, regression lines for each cell line were calculated (Fig. 4c) and subjected to analysis of covariance (see Methods). Compared with control shRNA-expressing T84 cells, the adjusted mean leakage rates were significantly lower for APP KD i1 cells (by 0.38 ± 0.12 µM/s, *p* = 0.0016) and APP KD i2 cells (by 0.61 ± 0.14 µM/s, *p* < 0.0001). Thus, the downregulation of *APP* expression increased resting [Ca^2+^]_ER_, which may be explained by the slower leakage of Ca^2+^ from the ER.

## Discussion

In the present study, we utilised two different types of ER-localised Ca^2+^ sensors to estimate Ca^2+^ levels in the ER in APP-deficient cells: a stably expressed GECI (GEM-CEPIA1er) and an endogenous ER Ca^2+^ sensor (STIM1). Ratiometric GECIs, such as GEM-CEPIA1er and D1ER, are advantageous for measuring resting ER Ca^2+^ levels in intact cells because they can be calibrated to obtain absolute [Ca^2+^]_ER_ values, and they do not require any cell manipulation prior to the measurements. The latter cannot be avoided when working with some other frequently used ER Ca^2+^ indicators, such as aequorin (which requires reconstitution with its cofactor) or the chemical probe MagFura-2 (which requires loading into cells and subsequent cell permeabilisation). Moreover, when using either of these two indicators, steady-state ER Ca^2+^ levels are recorded after store refilling and may not necessarily reflect physiological resting levels. As demonstrated in this work using GEM-CEPIA1er, T84 cells with downregulated *APP* expression had substantially elevated resting [Ca^2+^]_ER_ compared with APP-containing cells. Furthermore, they exhibited delayed translocation of the endogenous ER Ca^2+^ sensor STIM1 to the Ca^2+^ channel Orai1 upon CPA-evoked Ca^2+^ store depletion, reflected by the STIM1/Orai1 co-localisation coefficient. Although we cannot exclude a direct effect of APP on the translocation of STIM1, these two lines of experimentation suggest that the lack of APP results in an elevation of resting [Ca^2+^]_ER_, which in turn causes a delay in reaching the threshold for STIM1 oligomerisation and translocation upon Ca^2+^ store depletion. The advantage of the latter experimental method is that it does not perturb resting [Ca^2+^]_ER_ by the presence of exogenous indicators, which unavoidably act as Ca^2+^ buffers. The translocation of STIM1 to Orai1 in wildtype T84 cells was a relatively slow process, with half-maximal values reached at 6.3 min after the addition of CPA. The observed translocation kinetics was likely limited by the kinetics of store depletion. The inhibition of SERCA with CPA causes passive and slow Ca^2+^ release through poorly characterised “leak channels”^2^. This approach, however, was advantageous for our analysis because we sought to reveal differences in initial ER Ca^2+^ levels.

Interestingly, we did not observe any differences between APP-depleted and control cells in the co-localisation of STIM1 with Orai1 12 min after the addition of CPA (i.e., the time point when co-localisation reached saturation in wildtype cells). This suggests that the same amount of STIM1 binds Orai1 when given sufficient time for Ca^2+^ store depletion. This is consistent with our previous findings that APP had no effect on SOCE in cells with fully depleted Ca^2+^ stores^37^. Thus, the APP-dependent effect on SOCE that was observed by some authors (but not others; see discussion in Wegierski *et al*.^37^) may be merely a consequence of changes in ER Ca^2+^ content. A similar conclusion was reached regarding the observed effects of PS1 on SOCE^15,38^.

The involvement of APP in cellular Ca^2+^ homeostasis has most often been investigated using cytosolic Ca^2+^ indicators. As recently reported, Fura-2-loaded hippocampal neurons from APP knock-in mice (bearing two pathogenic mutations) exhibited enlarged ionomycin-sensitive intracellular Ca^2+^ stores^39^. This Ca^2+^ overload was proposed to result from persistent overactivation of mGluR5 receptors by Aβ_42_. In addition, wildtype APP was implicated in ER Ca^2+^ handling by Linde *et. al*. who demonstrated attenuation of CPA-evoked ER Ca^2+^ release in cortical astrocytes isolated from APP knockout (KO) mice compared with wildtype astrocytes^40^. In contrast, APP KO neurons had normal ER-releasable Ca^2+^ pools^41^ and knockdown of APP in neuroblastoma cells resulted in larger cytosolic Ca^2+^ transients upon SERCA inhibition^42^. It should be noted that such measurements with cytosolic indicators provide a net outcome of the activities of ER Ca^2+^ channels from one side and Ca^2+^ extrusion or buffering mechanisms from the other side. Therefore, the reported discrepancies may reflect not only differences in resting ER Ca^2+^ levels between different cell types but also differences in the activities of Ca^2+^ handling machineries. This emphasises the importance of using organelle-targeted Ca^2+^ probes to investigate organellar Ca^2+^ levels. However, evidence of a role for APP in ER Ca^2+^ handling that is supported by direct measurements of [Ca^2+^]_ER_ is scarce. Using ER-targeted aequorin, the overexpression of a FAD-causing APP_SWE_ mutant in neuroblastoma cells was found to reduce steady-state ER Ca^2+^ loads through accelerated ER Ca^2+^ release^28^. No data were provided for wildtype APP. In this work, we found that knocking down *APP* expression with either of two different *APP*-targeting shRNAs led to an elevation of resting ER Ca^2+^ levels in intact T84 cells (Fig. 4a), the prolonged emptying of ER Ca^2+^ stores upon SERCA inhibition (Fig. 4b), and delayed STIM1 translocation to Orai1 (Fig. 3c). Moreover, APP-deficient cells exhibited lower passive Ca^2+^ leakage rates out of the ER (Fig. 4c), which may at least partially explain the aforementioned observations. Taken together, our results suggest that APP is involved in the maintenance of physiological [Ca^2+^]_ER_, a prerequisite to normal ER Ca^2+^ signalling.

Up to date, several functional roles have been proposed for APP but the elucidation of underlying mechanisms has been hampered by the extensive proteolytic processing of APP, giving rise to fragments with different or even opposing roles^43^. Whereas secreted APPα is neuroprotective, Aβ exerts toxic effects on cells, including elevation of intracellular Ca^2+^ levels^43^. Because our study employed a loss-of-function approach, it did not resolve whether the normalisation of [Ca^2+^]_ER_ is mediated by full-length APP or one of its processing fragments. Although our findings contribute to the elucidation of physiological functions of APP, they may also be relevant to AD pathology. Dysregulation of ER Ca^2+^ signalling has been described in many cellular and animal models of AD, and proposed to underlie dendritic spine loss in neurons^39,44^. Our results are consistent with Oules *et al*.^28^ and suggest that both wildtype APP and the APP_SWE_ mutant similarly affect ER Ca^2+^. However, the two APP variants might confer different regulatory capabilities of ER Ca^2+^, possibly due to an altered balance between the amyloidogenic and non-amyloidogenic pathways.

Compared with APP, Ca^2+^-related functions of FAD-linked PSs have been studied more thoroughly. PS1 FAD mutations have been repeatedly shown to enhance Ca^2+^ release from the ER upon cell stimulation. Several mechanisms have been proposed by different research groups for PS1 FAD mutants to explain the enhancement of ER Ca^2+^ signalling, including: (*i*) increases in the levels and function of ryanodine receptors^45^, (*ii*) augmented IP_3_ receptor activity^17^, (*iii*) SERCA activation^46^, and (*iv*) the loss of leak channel activity^22^. However, none of these mechanisms gained broad acceptance, and some were questioned^21^. These studies usually also investigated the Ca^2+^-related functions of wildtype PSs compared with PS1 mutants. When the measurements were made with the D1ER indicator, PS double knockout (PS DKO) fibroblasts had elevated resting Ca^2+^ levels compared with wildtype fibroblasts, and this phenotype was rescued by the expression of wildtype PS1^20^. The same conclusions were reached when the measurements were made with MagFura-2-loaded and permeabilised PS DKO fibroblasts^22,24^. To explain these observations, wildtype PS1 was shown to act as a Ca^2+^-permeable ER leak channel itself^22^. This finding has recently gained further support by a study reporting the dual function of archeobacterial PS homologues as proteases and ion channels^47^. Furthermore, a screen for Ca^2+^ regulatory proteins identified PS2 as an important ER Ca^2+^ leak factor^48^. An alternative mechanistic explanation for the effects of PSs was provided by Brunello *et al*., who found that PS2 accelerated ER Ca^2+^ leakage through IP_3_- and ryanodine receptors and simultaneously inhibited SERCA2^16^. In addition, other data did not support the inverse relationship between wildtype PS1 levels and ER Ca^2+^ levels or the proposed mechanisms of action of wildtype PS1^18,21^. These discrepancies may be attributable to the different cell types and methodologies employed to measure ER Ca^2+^ dynamics.

In conclusion, the present findings indicate that wildtype APP, similar to PSs, may also be involved in regulating ER Ca^2+^ levels. In particular, we show that T84 cells depleted of APP exhibited elevated resting [Ca^2+^]_ER_ and slower passive ER Ca^2+^ leakage. We also demonstrate that differences in the resting levels of ER Ca^2+^ between cells can be estimated by measuring the translocation of endogenous STIM1 to Orai1 upon SERCA inhibition. Our results reveal a physiological function of APP in ER Ca^2+^ homeostasis, which may have important implications for AD research.

## Methods

### Reagents

ProLong Gold and Hoechst 33342 were purchased from Life Technologies. Ionomycin (free acid) was purchased from Calbiochem. CPA, ML-9, polybrene, poly-L-lysine (PLL), fetal bovine serum (FBS), puromycin, and penicillin/streptomycin were purchased from Sigma-Aldrich. Escin was purchased from Santa Cruz. YM-58483 and SKF-96365 were purchased from Abcam. The following antibodies (Abs) were used: anti-STIM1 (HPA012123), anti-SERCA2 (S1439), anti-β-actin (A5441) were from Sigma-Aldrich, anti-APP (Y188) was from Abcam, and anti-Orai1 (G2; sc-377281) was from Santa Cruz.

### Nucleotide sequences and plasmids

The following shRNAs were designed using the Whitehead Selection Web Server^49^: APP KD i1 (GAAGGCAGTTATCCAGCAT), APP KD i2 (GGTGCAATCATTGGACTCA), STIM1 KD (GAAAGTGATGAGTTCCTGA), ORAI1 KD i1 (GCAACGTGCACAATCTCAA), ORAI1 KD i2 (GTGTGTGTGACACATAAAT), and SERCA2 KD (GCAACTCAGTCATTAAACA). Control non-targeting shRNA was CCTAAGGTTAAGTCGCCCT. The shRNAs were cloned between EcoRI and ClaI sites into modified pLVTH vectors (originating from Addgene plasmid no. 12262), in which GFP cDNA was replaced with the GEM-CEPIA1er sequence (originating from Addgene plasmid no. 58217), or both GFP cDNA and the EF1α promoter were replaced with a puromycin selection marker and PGK promoter. DsRed2-ER cDNA was cloned into a pQCXIP retroviral vector (Clontech).

### Cell cultures, virus production, and transduction

All cells used in this study were obtained from ATCC. HEK293T/17, HeLa, and U-87 cells were grown in Dulbecco’s modified Eagle’s medium (DMEM), Jurkat cells were grown in RPMI-1640, and T84 cells were grown in DMEM/F-12 supplemented with 15 mM HEPES. All media contained 10% FBS and 1% penicillin-streptomycin. All gene knockdowns (KD) were performed by transducing cells with lentiviruses that carried targeting shRNA sequences and the puromycin selection marker or the GEM-CEPIA1er sequence. Viruses were prepared in 293 T/17 cells using the Ca^2+^-phosphate transfection method. Supernatants were collected 48–72 h after transfection, filtered through 0.45-µm membranes and concentrated in Vivaspin 100-kDa units (Sartorius) in a swing-out rotor at 1,000 × *g*. The viral titers were ~10^6^ transducing units per ml. T84 and HeLa cells were transduced in the presence of 8 µg/ml polybrene for 8 h. T84 cells transduced with viruses carrying the puromycin marker were selected with 5–10 µg/ml puromycin for 2 weeks. Experiments with these cells started at least one week after puromycin withdrawal. Experiments with cells expressing GEM-CEPIA1er were conducted between 1–2 weeks after transduction. The ER localisation of GEM-CEPIA1er in APP KD and control T84 cells is presented in Supplementary Fig. S3.

### Immunofluorescence and co-localisation analysis

T84 cells were seeded on PLL-coated coverslips. The next day, they were transferred to Ringer solution (150 mM NaCl, 6 mM KCl, 1 mM MgCl_2_, 10 mM glucose, and 10 mM HEPES, pH 7.4) supplemented with 1.8 mM CaCl_2_. To deplete ER Ca^2+^ stores, the cells were washed in Ringer solution supplemented with 0.5 mM EGTA, and incubated for defined periods of time in Ringer solution that contained 0.5 mM EGTA and 30 μM CPA. The incubations were carried out at room temperature. In some experiments, SOCE inhibitors (50 µM ML-9, 30 µM SKF-96365, or 10 µM YM-58483) or DMSO (drug vehicle) were added to the CaCl_2_-containing Ringer solution (pretreatment of cells for 5 min) and also to the EGTA and EGTA/CPA solutions (CPA treatment). For experiments presented in Fig. 2d, Ringer solution contained either 10 mM CaCl_2_ (as in high-Ca^2+^ medium) or 1.8 mM CaCl_2_ (normal medium control) before starting ER Ca^2+^ store depletion. After treatments, the cells were fixed in 3.7% paraformaldehyde in PBS, permeabilised in 0.1% Triton X-100 and 0.05% SDS in PBS, and stained against endogenous STIM1 and Orai1 proteins. Nuclei were stained with Hoechst 33342. The cells were then mounted in ProLong Gold. Images were captured under Zeiss LSM5 Exciter or Zeiss LSM800 confocal microscope using a Plan-Apochromat 63×/1.4 oil objective. Acquired cells were chosen based exclusively on Hoechst staining. All of the images were batch processed using custom-written scripts in ImageJ software. Mander’s M1 colocalisation coefficient^50^ was calculated with the Manders Coefficients ImageJ plugin. To obtain the extent of specific co-localisation of STIM1 with Orai1 (referred to as “M1 STIM1/Orai1” in figures) for each image pair, co-localisation coefficients were first calculated for images that were displaced vertically or horizontally by 2.35 µm in each direction, and their mean value was subtracted from the co-localisation coefficient that was calculated for aligned images. The results were imported into Microsoft Excel software for statistical analysis.

### Ca^2+^ measurements

T84 cells were seeded on PLL-coated coverslips. Measurements of resting ER Ca^2+^ levels were performed the next day in cells incubated in Ringer solution (see above) supplemented with 1.8 mM CaCl_2_ under a Zeiss LSM800 confocal microscope with an EC Plan-Neofluar 40×/1.3 oil objective at room temperature. GEM-CEPIA1er signals were excited with a 405 nm laser, and the fluorescence light at wavelengths of 410–470 nm and 510–700 nm emitted by the Ca^2+^-bound and Ca^2+^-free indicator, respectively, was passed through a partially opened pinhole (460 µm) and simultaneously collected using two GaAsP detectors. 4–6 images from one field of view were acquired at 10 s intervals. An example of fluorescence intensity and ratio traces together with a calibration procedure is shown in Supplementary Fig. S4. The measurements of passive ER Ca^2+^ leakage were performed in cells continuously perfused with Ringer solution that contained either 1.8 mM CaCl_2_ or 0.5 mM EGTA and 30 μM CPA. Perfusion was driven by syringe pump 33 (Harvard Apparatus) at 1–2 ml/min. Images were acquired at 5 s intervals. At the end of each measurement, the cells were permeabilised in 150 μM escin for 2 min, and R_min_ and R_max_ values were obtained in the presence of 10 μM ionomycin under Ca^2+^-free and Ca^2+^-saturating conditions, respectively. The signals were calibrated as described previously^29^. All of the images were processed using ImageJ or MetaFluor software. The Ca^2+^ leakage rates were calculated over a 60 s time-window from the start of [Ca^2+^]_ER_ decline using the SLOPE function in Excel and plotted against initial [Ca^2+^]_ER_.

### Western blot

Lysates were prepared as described previously^37^. Proteins were separated on 7–12% SDS-PAGE using Tris-glycine buffer, and visualized by ECL reaction as described previously^37^. Uncropped blots are presented in Supplementary Fig. S5.

### Statistical analysis

ER Ca^2+^ leak rates were subjected to analysis of covariance as described^51^ using the StatTools web server (http://www.obg.cuhk.edu.hk). This analysis included the initial [Ca^2+^]_ER_ as a covariate. In short, the regression slopes for all cell lines (Fig. 4c) were analysed and found to be significantly different from zero. In the next step, the slopes were proven not to be significantly different from each other. Finally, the calculated differences in the mean leakage rates adjusted for the covariate were tested statistically (reported were: the difference to control cells, its error, and *p* value). The differences in the adjusted means are differences in leakage rates between the APP KD and control cells having the same initial [Ca^2+^]_ER_.

Other data were analysed using Excel or GraphPad Prism software using two-tailed unpaired or one-sample *t*-tests. The co-localisation data (*n* = 3–6) with calculated mean values and standard errors are presented in aligned dot plots. One *n* for the co-localisation analyses is defined as the mean result of six image pairs collected from an independent experiment. The calculated mean values of the Ca^2+^ measurements (n ≥ 6) are shown as bars with standard errors. One *n* for the Ca^2+^ measurements is defined as the mean result of all cells within one field of view, with the exception of the data presented in Fig. 4b,c, in which one *n* indicates one ER region. Values of *p < *0.05 were considered statistically significant.

### Data Availability

All data generated or analysed during this study are included in this published article and its Supplementary Information files.

## Electronic supplementary material

Supplementary Information
