# Supplementary material for: Knockdown of amyloid precursor protein increases calcium levels in the endoplasmic reticulum
Source: Sci Rep. 2017 Nov 6;7:14512. doi: 10.1038/s41598-017-15166-2 (PMC5673940; doi:10.1038/s41598-017-15166-2)
Supplement: Supplementary file 1 — Supplementary Information [file 41598_2017_15166_MOESM1_ESM.pdf]

Supplementary Information

Knockdown of amyloid precursor protein increases calcium levels in the endoplasmic reticulum

Kinga Gazda, Jacek Kuznicki & Tomasz Wegierski

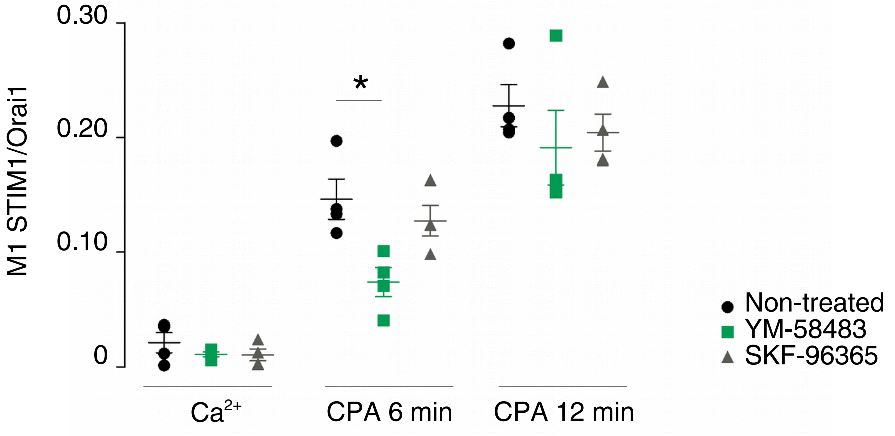

Supplementary Figure S1. The effect of SOCE inhibitors on the translocation of STIM1 to Orai1. Wildtype T84 cells, non-treated or pretreated with the SOCE inhibitors SKF-96365 (30 μM) or YM-58483 (10 μM) were fixed before (Ca^{2+}), 6 min after, or 12 min after the addition of 30 μM CPA in Ca^{2+}-free buffer. Fixed cells were stained with STIM1 Ab and Orai1 Ab. Aligned dot plots show calculated mean coefficients of co-localisation of STIM1 with Orai1 (M1 STIM1/Orai1) ± SEM (n = 4). Note that for SKF-96365-treated cells only 3 data points are visible for each time-point due to overlapping results. Differences between cells treated with the SOCE inhibitors and non-treated cells were analysed using unpaired t-tests (*$p < 0.05$).

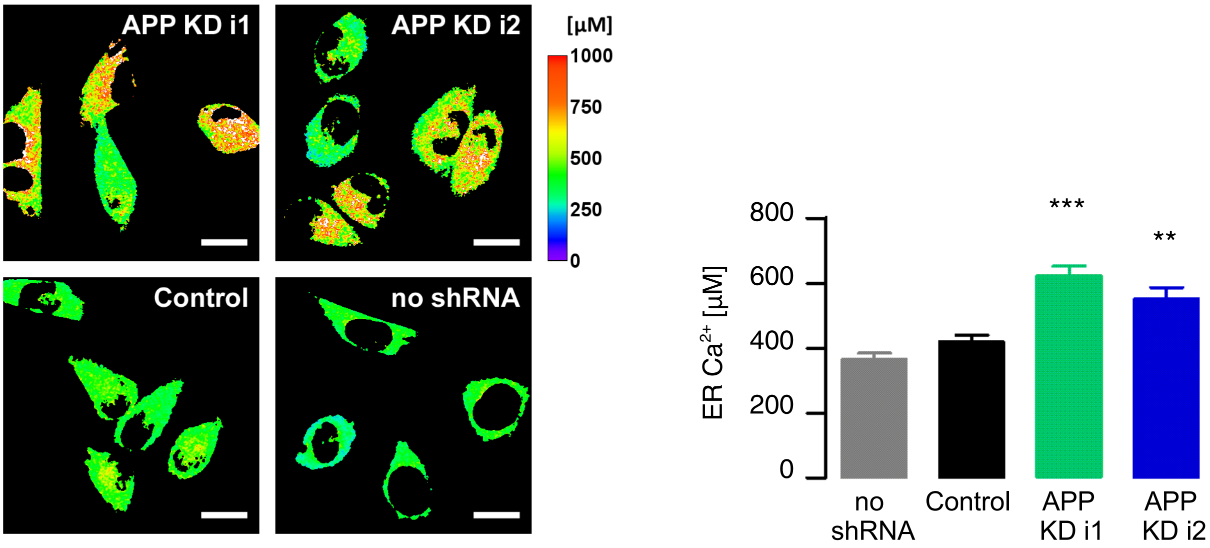

Supplementary Figure S2. APP-deficient HeLa cells have elevated resting levels of ER Ca^{2+}. The measurements of ER Ca^{2+} concentrations were conducted in HeLa cells after transduction with lentiviruses that carried nucleotide sequences coding for the ER Ca^{2+} indicator GEM-CEPIA1er and APP-targeting shRNA (APP KD i1 or APP KD i2; n = 11 and 12, respectively), control shRNA (Control; n = 12), or an empty shRNA cassette (no shRNA; n = 12). Images show representative heat maps of ER Ca^{2+} concentrations in the analysed cells (scale on the right). Scale bar = 20 µm. Mean ER Ca^{2+} concentrations are shown as bars with standard errors. Differences from control cells were analysed using unpaired t-tests (**$p < 0.01$, ***$p < 0.001$). Measurements were performed as described for T84 cells in Methods, except that images were acquired under a Zeiss LSM710 confocal microscope with a 20x/0.75 dry objective, the emission light at wavelenghts of 420−470 nm and 510−600 nm was passed through a pinhole opened at 196 µm, and the cells were permeabilised with 100 µM escin for 30 s before recording R_{min} and R_{max} values.

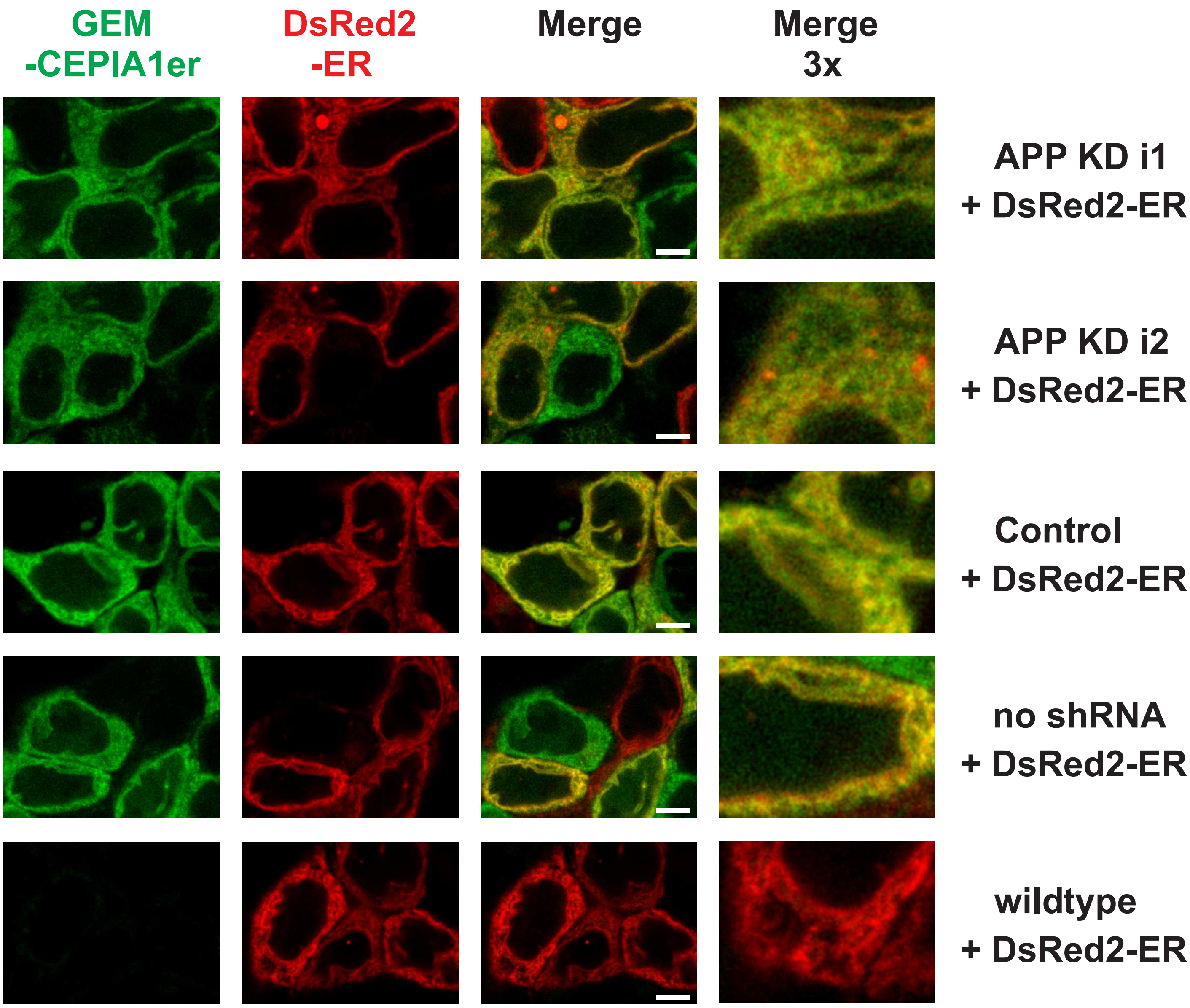

Supplementary Figure S3. GEM-CEPIA1er localises to the ER in T84 cells. Wildtype T84 cells and T84 cells stably expressing the Ca^{2+} indicator GEM-CEPIA1er and *APP*-targeting shRNA (APP KD i1 or APP KD i2), control shRNA (Control), or an empty shRNA cassette (no shRNA) were transduced with retroviruses encoding the ER fluorescent marker DsRed2-ER. GEM-CEPIA1er signals are shown in green and DsRed2-ER signals are shown in red. Yellow in the merged images indicates co-localisation between the two fluorescent proteins (magnified regions are shown on the right). Scale bar = 5 µm. Because GEM-CEPIA1er signals are very dim after fixation, imaging was performed on live cells incubated in Ringer solution containing 1.8 mM $CaCl_2$ at room temperature. Images were acquired under a Zeiss LSM800 confocal microscope using a 63×/1.4 oil objective. Pinhole was close to 1 Airy unit (55 µm). The fluorescent proteins were excited with a 405 nm laser (GEM-CEPIA1er) and with a 561 nm laser (DsRed2-ER) in separate acquisition tracks switched every line.

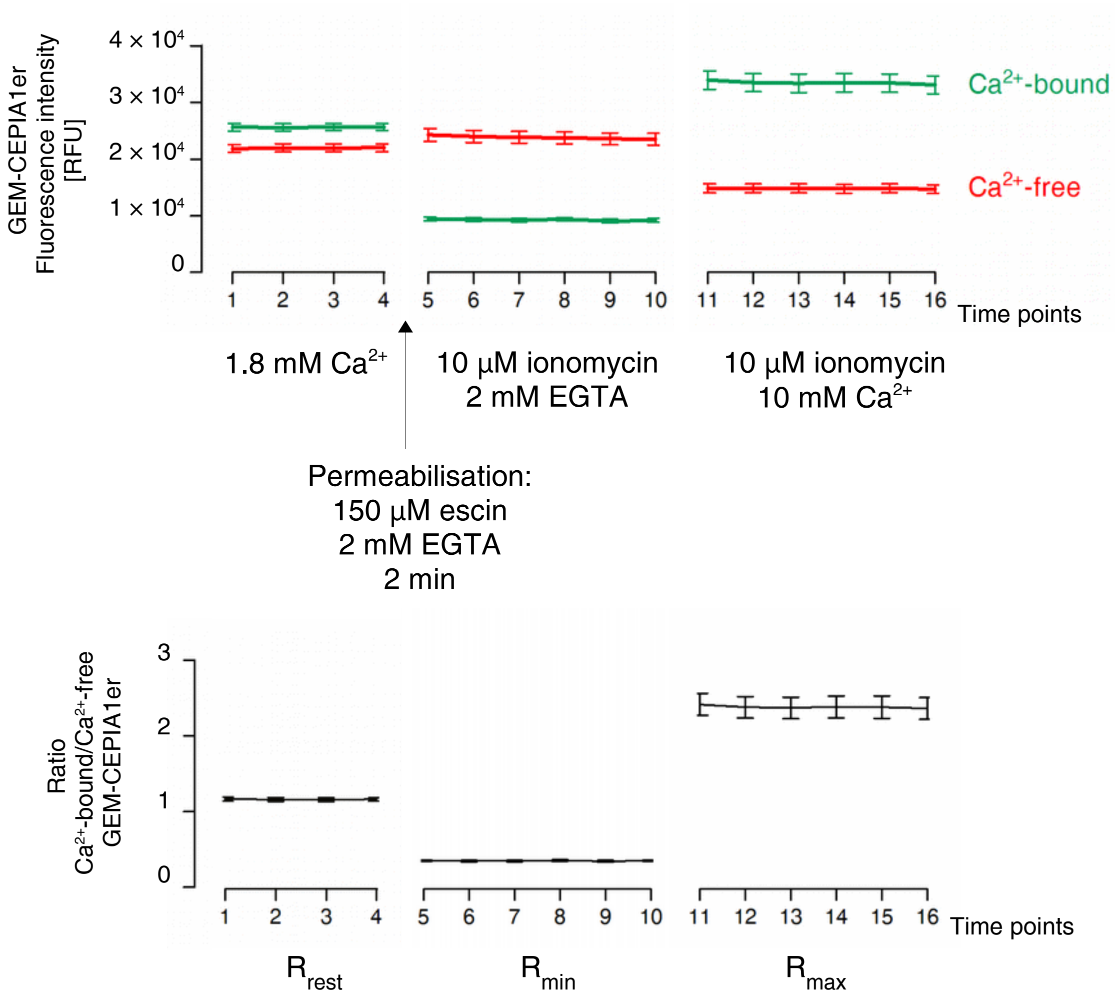

Supplementary Figure S4. An example of fluorescent intensity traces, ratio traces, and calibration procedure of the GEM-CEPIA1er signals in unstimulated T84 cells. The data derive from control cells (grown in DMEM/F12 media) that were used in the analysis presented in Fig. 2d. For clarity, mean traces ± SEM of all ER regions are shown rather than individual traces. Background-subtracted fluorescence intensities of Ca^{2+}-bound (green) and Ca^{2+}-free (red) GEM-CEPIA1er are shown as relative fluorescence units (RFU). Ratio (R) is the intensity of Ca^{2+}-bound GEM-CEPIA1er signal divided by the intensity of Ca^{2+}-free GEM-CEPIA1er signal. Microscopy imaging was performed as described in Methods. After R values were obtained in unstimulated cells (R_{rest}), the cells were permeabilised in 150 µM escin for 2 min (marked with an arrow), and R_{min} and R_{max} values were obtained under Ca^{2+}-free (2 mM EGTA and 10 µM ionomycin) and Ca^{2+}-saturating (10 mM $CaCl_2$ and 10 µM ionomycin) conditions, respectively. The values of R_{rest}, R_{min} and R_{max} were time-averaged for each ER region. Ratios were calibrated as described[29] using the equation: $[Ca^{2+}]_{ER} = [(R_{rest} - R_{min})/(R_{max} - R_{rest})]^{1/n} \times K_d$, where $n = 1.37$ and $K_d = 558$ µM.

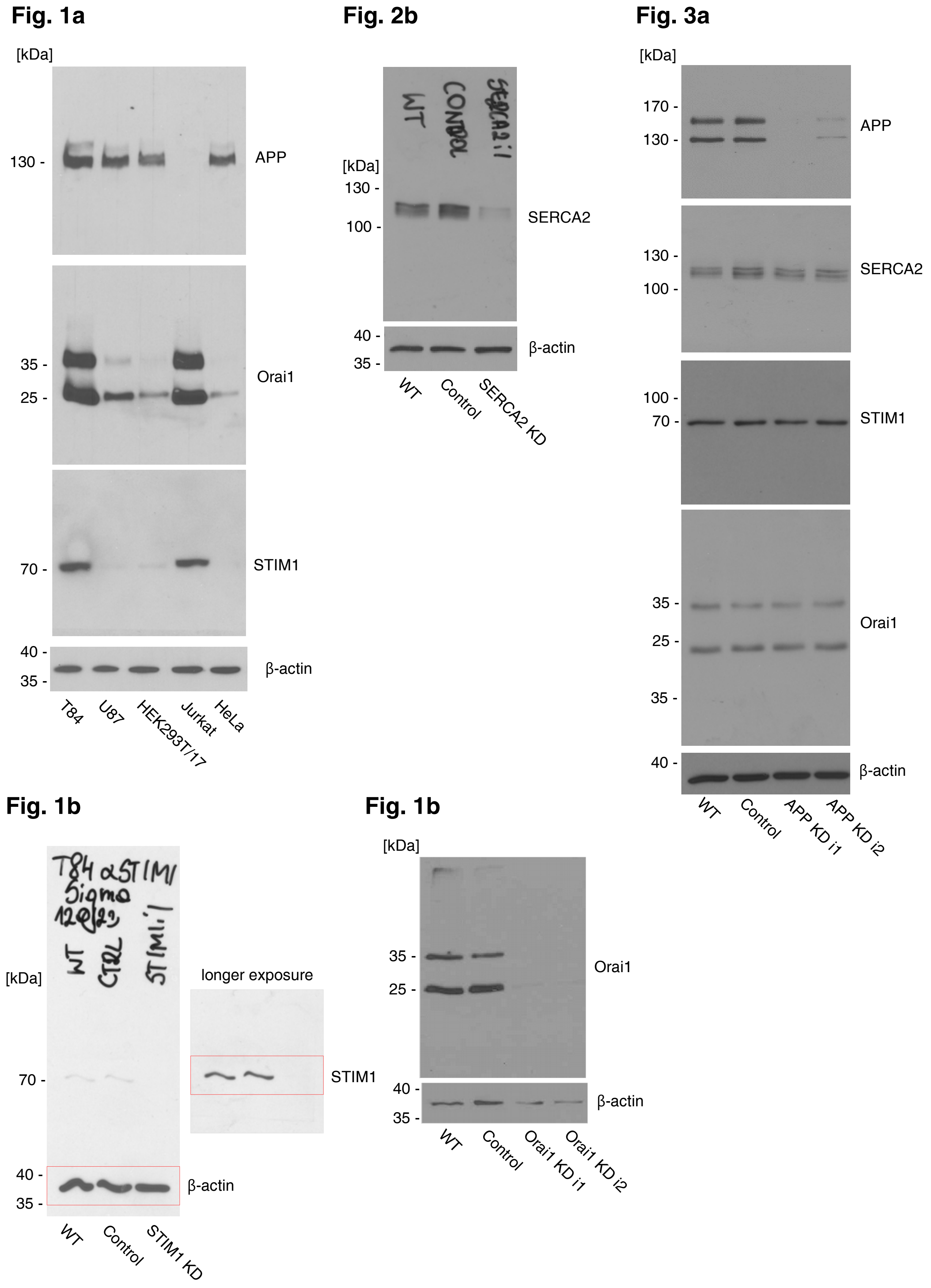

Supplementary Figure S5. Uncropped images of immunoblots shown in Fig. 1, Fig. 2, and Fig. 3.
